# Synthesis and Biological Application of Polylactic Acid

**DOI:** 10.3390/molecules25215023

**Published:** 2020-10-29

**Authors:** Ge Li, Menghui Zhao, Fei Xu, Bo Yang, Xiangyu Li, Xiangxue Meng, Lesheng Teng, Fengying Sun, Youxin Li

**Affiliations:** School of Life Sciences, Jilin University, Changchun 130012, China; lige18@mails.jlu.edu.cn (G.L.); zhaomh18@mails.jlu.edu.cn (M.Z.); xufei19@mails.jlu.edu.cn (F.X.); yangb202020@163.com (B.Y.); lixiangyu19@mails.jlu.edu.cn (X.L.); mxx18@mails.jlu.edu.cn (X.M.); tenglesheng@jlu.edu.cn (L.T.)

**Keywords:** PLA materials, drug delivery, polymer, implants, L- and D-lactic acid

## Abstract

Over the past few decades, with the development of science and technology, the field of biomedicine has rapidly developed, especially with respect to biomedical materials. Low toxicity and good biocompatibility have always been key targets in the development and application of biomedical materials. As a degradable and environmentally friendly polymer, polylactic acid, also known as polylactide, is favored by researchers and has been used as a commercial material in various studies. Lactic acid, as a synthetic raw material of polylactic acid, can only be obtained by sugar fermentation. Good biocompatibility and biodegradability have led it to be approved by the U.S. Food and Drug Administration (FDA) as a biomedical material. Polylactic acid has good physical properties, and its modification can optimize its properties to a certain extent. Polylactic acid blocks and blends play significant roles in drug delivery, implants, and tissue engineering to great effect. This article describes the synthesis of polylactic acid (PLA) and its raw materials, physical properties, degradation, modification, and applications in the field of biomedicine. It aims to contribute to the important knowledge and development of PLA in biomedical applications.

## 1. Introduction

The progress and optimization of high polymer materials play an important role in industrial production, especially in the fields of electronics, chemical and medical treatment. Since the synthesis of artificial plastics in the early 20th century, plastic polymer materials have been used in different fields and subject to significant development. At first, chemical polymers produced in the petroleum industry were favored due to their low cost, ease of production, excellent mechanical properties, and good heat sealability. Their rapid production had a negative impact on resources, economies, the environment, and safety, for example, (a) the use of oil and gas resources has led to increases in the prices of oil and natural gas; (b) the progress of global environmental warming; (c) the use of nonrecyclable materials; and (d) the occurrence of cross-contamination. Toxicity risk results from consumers’ inability to metabolize their monomers [1,2]. That is to say, in the natural environment, it is not easy for plastics to spontaneously be completely degraded, especially in the face of large-scale use of plastic film coverings and packaging, which generate a large amount of waste and cause serious pollution and damage to the ecosystem [3]. Under the destruction of the environment due to the development of industry, the threat to human health is enormous, and millions of dollars are spent every year to deal with it. Therefore, research and development in the field of renewable, biodegradable plastics is attracting increasing attention from researchers. Biodegradable materials can gradually and spontaneously degrade in the natural environment, lastly entering nature in the form of small molecules. Polylactic acid (PLA) is a kind of lactic acid (LA) derivative produced from renewable resources such as wheat, straw, corn, and sorghum, and it is completely biodegradable [4,5]. It is environmentally friendly, and can be decomposed into water and carbon dioxide by microbes. Given the problem of global pollution, focusing on improving the environment is a win–win situation; thus, it is considered the most promising biodegradable polymer material on the market [6,7]. Although lactic acid, the monomer of polylactic acid, can be easily obtained in nature, it is traditionally produced by the fermentation of sugars and conversion into monomers after hydrolysis [8]. However, while a variety of synthetic methods have been studied for industrial and commercial use, none of these is simple, easy to operate, or economical. There are strict requirements with respect to the catalysts, synthesis steps, and purification equipment used in the synthesis process [9]. Fortunately, a moderate way is provided by biological methods using enzyme-catalyzed synthesis processes, which make biocompatible materials more readily available than drastic chemical reactions. In terms of nature, polylactic acid possesses good thermoplasticity and can be used to prepare and process plastics in order to make, for example, films and fibers [10,11]. Polylactic acid is not easily affected by solvent swelling and dissolution during industrial processing. Processing temperature is generally 170–230 °C, so it is also suitable for extrusion, spinning, biaxial stretching, and injection blow-molding processing methods [12,13,14]. Simultaneously, polylactic acid material has good biocompatibility, biodegradability, gloss, and transparency, and antibacterial, flame-retardant, and oil- and water-resistant properties. It has a very wide range of uses, such as in packaging materials and nonwoven fabrics, and in the clothing, manufacturing, and healthcare industries [15,16,17,18,19]. At first, it is used in medical sutures and bone screws because of these properties. The ester bonds of the polymer backbone are broken by hydrolysis without additional surgeries, and the degradation products are non-toxic and avoid part of the immune response, which make it an ideal candidate for biomedical applications. The promising development of nanomedicine benefits from the synthesis of nanomaterials. The synthesis of nanomaterials provides a good prospect for the development of nanomedicine. Nano-polymers can be used to encapsulate chemical drugs, proteins, and genetic drugs to enhance the circulation of drugs in the human body while reducing the toxic side effects of drugs. Polylactic acid occupies an indispensable position in modern industrial technology. This article focuses on the synthesis, properties, and application of modified polylactic acid in the biomedical field.

## 2. PLA Synthesis and Properties

### 2.1. Lactic Acid

**Figure 1:**
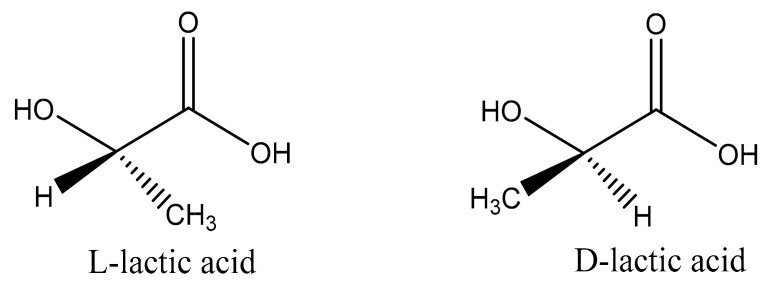
Enantiomers of lactic acid.

Lactic acid is a monomer that comprises polylactic acid, and its existence is very common. It plays a vital role in the glycolytic energy cycle in organisms, as well as being an important substance for maintaining the growth and development of living organisms [20]. LA was first extracted from fermented milk by Swedish scientists in 1881. As the simplest hydroxy acid, there is an asymmetric carbon atom in the molecule that is optically active, so there are two optical isomers, l-lactic acid and d-lactic acid, as shown in Figure 1 [21,22]. Lactic acid obtained from animal muscle is dextrorotatory lactic acid, while the other, lactic acid obtained by fermentation, is levorotatory lactic acid. In the beginning, commercial lactic acid was mainly derived from the fermentation of sugars by bacteria [23]. Sugars used for lactic acid fermentation include starch, glucose, lactose, and maltose, produced by corn and potatoes. Fermentation is typically carried out for 3–5 days under low pH (about 5.0) and low oxygen at a temperature of about 40 °C [24]. However, this method produces a certain degree of toxicity as the concentration of fermented lactic acid increases, and the reaction continues. To obtain high-purity LA and improve the output efficiency of lactic acid, purification also constitutes a major problem [25]. In the beginning, the typical purification method was to add calcium hydroxide or calcium carbonate to the fermentation broth to neutralize the acid. The resulting calcium lactate could be evaporated, crystallized, and acidified to obtain further crude lactic acid and insoluble calcium sulfate (gypsum). Lactic acid used in food or medicine often needs to be further purified to obtain a higher degree of lactic acid. Subsequent studies showed that it can then be neutralized with recycled ammonia or sodium hydroxide. Subsequently, ultrafiltration, dyeing, and electrodialysis were developed as new processes for the low-cost production of lactic acid [26].

Chemical synthesis methods have also been applied for the industrial production of lactic acid. These fall into roughly three types: (a) The lactonitrile method. Lactonitrile is hydrolyzed by sulfuric acid to obtain crude LA. The crude LA is added to ethanol for esterification, and refined lactic acid is obtained by distillation, concentration, and decomposition. (b) The acrylonitrile method. Like the above method, but with acrylonitrile replacing lactonitrile, hydrolyzed with sulfuric acid. Then, the hydrolyzed product is reacted with methanol; the crude ester is sent to the distillation tower, and the refined ester is heated and vacuumed to obtain the product. (c) The propionic acid method. Using propionic acid as raw material, crude lactic acid is obtained through chlorination and hydrolysis; then, the product is obtained through esterification, rectification, and hydrolysis. Only a few manufacturers use this method due to the expensive raw materials [27].

Although chemical methods are able to achieve large-scale continuous production of LA, and the U.S. Food and Drug Administration (FDA) has approved the resulting product, the raw materials are generally toxic. They do not accord with the standards of green chemistry. Some recent studies showed that, by adding bacteria and substrate-degrading enzymes for simultaneous saccharification and fermentation, starch or cellulosic biomass can be saccharified and simultaneously converted into lactic acid; this process can be used as a replacement for the above-mentioned traditional fermentation steps [28,29]. This process reduces costs and improves efficiency.

### 2.2. PLA Polymer

Since LA is a chiral molecule with d-type and l-type isomers, three forms of polylactic acid are formed: poly-l-lactic acid (PLLA), poly-d-lactic acid (PDLA), and poly-d,l-lactic acid (PDLLA). With respect to optical activity, there are two compositions-L and D enantiomers-such that polylactic acid can be crystallized into three forms (α, β, and γ) [30,31,32]. In 1932, Carothers (DuPont) synthesized low-molecular-weight polylactic acid products. However, due to the high cost of synthesis and poor product stability, this synthesis was not considered a success [33]. In 1954, DuPont produced a higher-molecular-weight polylactic acid, and applied for a patent intending to enter the commercial market. Research on polymers attracted significant attention from society. With advances in medicine and public health, polylactic acid began to be used in surgical sutures and bone implants in the 1960s. Today, PLA resin has been approved by the FDA and European regulatory authorities for use in food- and drug-delivery systems [12,34]. This has also led people to realize that, while PLLA has some advantages, it also has some disadvantages. For example, due to its high crystallinity and slow degradation rate, it can trigger an inflammatory response in the body. Fortunately, d-lactic acid degrades more quickly. If l-lactic acid and d,l-lactic acid monomers are used to synthesize PLLA, the above problems are avoided [35,36].

**Figure 2:**
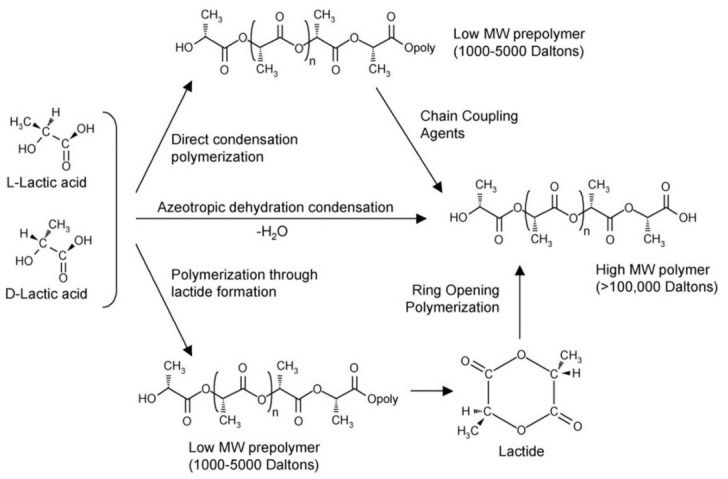
Synthesis of polylactic acid (PLA) from l- and d-lactic acids [28]. Adapted from Auras et al. by permission of Wiley–VCH Verlag GmbH & Co. KGaA.

Three methods for the synthesis of polymer PLA (Mw > 10,000) were reported: (a) direct condensation polymerization; (b) azeotropic dehydration condensation; and (c) lactide ring-opening polymerization, as shown in Figure 2.

Although the cost of the condensation-polymerization method is low, it cannot directly synthesize polymer PLA. Rather, part of the cost corresponds to the coupling agent and esterification accelerator used in the synthesis process. The synthesis step is divided into two steps [37,38,39]. The first step is the dehydration condensation of hydroxyl and carboxyl groups at equimolar concentrations to produce low-molecular-weight polylactic acid. Next, coupling agents and esterification adjuvants need to be added. Their addition can modify the PLA and help amplify the chain. However, impurities in the reaction process cannot be degraded in the body, which can cause serious complications in medical applications [40]. To obtain a high-purity, low-molecular-weight oligomer final product with no residual metal or catalyst, it is necessary to introduce triphosgene to remove the adjuvant and byproducts in the reaction. However, in addition to higher economic costs, this method uses flammable solvents, thus increasing safety risks. Although later studies found that new chain extenders could replace the esterification of the above-mentioned adjuvants, there is still the problem that chain extenders and polymer impurities are toxic and nonbiodegradable [41]. Although the azeotropic dehydration condensation method avoids the use of adjuvants during the synthesis of PLA, the disadvantage of this method is that dibasic acids and glycols are used as solvents in the reaction while the catalyst remains. First, lactic acid is distilled under reduced pressure at 130 °C for 2–3 h to remove most condensed water. The catalyst and diphenyl ether are added to the reaction. This is passed through a molecular sieve and returned to the container for another 30–40 h at a temperature of 130 °C. The polymer can then be separated or dissolved and precipitated for further purification. In the subsequent production process, due to the effective removal of water, the increase in the boiling point of the solvent results in an increased polymerization rate. After testing a variety of catalysts, it was found that tin compounds have higher catalytic efficiency. In addition, the content of impurities hinders the synthesis to a certain extent. In follow-up industrialization research, it was demonstrated that it is possible to remove the catalyst to a great extent without degrading the polymer. However, the toxicity and nonbiodegradability of the remaining catalyst can cause irreversible damage to the human body, so it cannot be applied in the medical field.

**Figure 3:**
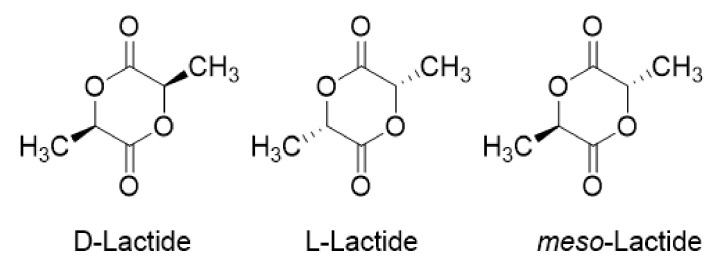
Stereoforms of lactides.

The ring-opening polymerization of lactide is one of the methods for industrial production of high-molecular-weight PLA. Lactide has three stereo configurations—l-lactide, mesolactide, and d-lactide—as shown in Figure 3 [42]. As a cyclic dimer, it can be formed by solvent-free dehydration under mild conditions. Commercially feasible methods for obtaining and purifying lactide involve steps such as condensing lactic acid at 115–179 °C, removing the condensed water, and removing the mesolactic acid and low-molecular polymer through recrystallization to obtain pure l- or d, l-lactide with high molecular weight [9,43,44]. The industrial production method of lactide is the same as the scheme mentioned above, but in different reactors, producing low-molecular-weight prepolymers, and the final purification method is different. For example, Cargill Inc. uses a reduced pressure–reflux method to remove residual water, lactic acid, oligomers, and partial lactide [45]. Nemphos changed the purification step, using a multistage melt recrystallizer to remove LA and low-molecular polymer. Bhatia and colleagues used inert gas to remove, recrystallize, and purify lactide. Other methods include the use of lactide to gas-phase recrystallization to increase yield and weakly alkaline water/solvent systems to extract lactide. In the method described above, after removing impurities, high-purity lactide is generally obtained.

After obtaining high-purity lactide, depending on the catalyst, the ring-opening polymerization of lactide can adopt one of three mechanisms: cation, anion, and coordination/insertion. Cationic initiators can generally be divided into protic acid, Lewis acid, and alkylating or acylating reagents. Kricheldorf and his colleagues found that triflic acid and methyl triflate, among many cationic initiators, could effectively induce the polymerization of lactide. The choice of different anion inducers causes deprotonation, resulting in inconsistent polymerization and racemization, leading to polymers with different molecular weights [46]. Considering that metal ions can cause toxicity problems, it is not recommended to use butyl lithium or crown ether initiators. The use of primary alkoxides, 6-valerolactone, or polyethylene glycol can produce clearly defined polymers. The two above methods have high reactivity, and are usually prone to racemization and transesterification during the solvent reaction process, resulting in impurities. The use of metal carboxylates, oxides, and alkoxides with lower activity to produce polylactide with low toxicity and few impurities has been extensively studied in commercial production applications. Studies found that the use of tin (II) and zinc produces fewer impurities during the synthesis of high-molecular-weight polylactic acid, resulting in the purest polylactic acid. For example, tin (II) di-2-ethyl hexanoic acid has high catalytic activity and low toxicity. It was approved by the FDA, and it is a highly suitable inducer. In addition to tin compounds, aluminum alkoxides and rare-earth compounds are other catalyst systems that proceed through coordination/insertion mechanisms. Research found that the polymerization rate of rare-earth compounds is still much higher than that of aluminum alkoxides.

Additionally, according to research, enzymatic polymerization is more environmentally friendly than chemical synthesis methods are. Enzymatic reactions require a mild environment, and enzymes are efficient, inexpensive, and have high specificity. Chemical reactions require a single reactant to avoid side reactions. However, not many references are available for this method [47].

### 2.3. PLA Properties

Polylactic acid has very good market prospects and excellent commercial value, with a range of applications from industrial to civilian use. Interest in its mass production stems from its good performance; for example, polylactic acid has good physical properties and can be used to yield various plastic products, such as fast-food lunch boxes, and fabrics for industrial and civilian use [44,48,49,50]. Good tensile strength and ductility make it suitable for different processing means, such as melt-extrusion molding, injection molding, blown film molding, foam molding, and vacuum molding. Its good biocompatibility has led it to be widely used in the field of biochemical medicine. High-molecular-weight PLA has been used to produce non-dismantling surgical sutures and low-molecular-weight PLA as a slow-release drug-packaging agent [51].

Polymer polylactic acid possesses good characteristics with regard to gloss, transparency, hand feel, and heat resistance. The dissolution of PLA depends on its degree of crystallinity. Amorphous polymers are soluble in various organic solvents, including acetone, acetonitrile, and methylene chloride. Furthermore, crystalline PLA can only be dissolved in dichloromethane or benzene at high temperatures. Mechanical properties and crystallization behavior are closely related to the molecular weight and stereochemical composition of the main chain. The hydroxy compound determines the molecular weight. The degree of crystallinity affects the degradation of PLA [52,53]. Highly crystalline polymers last for several months, with metabolism only taking place after a few years, while polymers with low crystallinity can be decomposed in a few weeks. Glass transition (Tg) directly affects the performance of the material with respect to both use and processing [54]. The good news is that the crystalline state does not affect the glass transition temperature. Jamshidi’s research found that the glass transition temperature of PLA with a molecular weight of 22,000 g mol^−1^ was 55 °C, which is only 5 °C away from polymers of infinite molecular weight. Impact resistance increases with increasing crystallinity and molecular weight. Pure PDLA and PLLA have a melting point (Tm) of 207 °C. When they are mixed in equal proportions, racemic crystals with better mechanical properties are formed. The Tm of this crystal is 230 °C, and ultimate tensile strength is 50 MPa. Tg and Tm are vital physical parameters with respect to the properties of polylactic acid. The heat of fusion (△Hm) of 100% pure crystals is 93.7 J g ^−l^. Subsequent research results showed that, with low polylactic acid content, the Tm and Tg of polylactic acid are decreased.

Polylactic acid has sufficient thermal stability to retard degradation and maintain molecular weight and performance. At temperatures greater than 200 °C, polylactic acid undergoes hydrolysis, lactide recombination, oxidative main-chain scission, and intermolecular or intramolecular transesterification.

The degradation of polylactic acid is contingent on many factors, including time, temperature, low-molecular-weight impurities, and catalyst concentration [27,55,56]. Studies found that simply modifying the purified pure polymer affects degradation. For example, acetylation of the hydroxyl end increases the decomposition temperature by 26 °C and ameliorates the decrease in molecular weight. At present, there is a general theory that the phenomenon resulting in PLA degradation is the process of simple proton-catalyzed hydrolysis chain scission [57]. Since the reaction is reversible, the purity of the polymer affects the degradation process during the synthesis reaction process, that is, the purity of the polymer can be used to explain the degradation kinetics of PLA. Furthermore, the crystallinity of the compound, as described above, determines the degradation rate and autocatalysis. When PLA is hydrolyzed and degraded in a biological environment, enzymes also participate. However, the specific role of enzymes in the reaction is currently unclear. Whether the reaction is catalyzed directly or by removing byproducts, so that the reaction is beneficial to forward progress, is not known. Supposing polylactic acid is mainly degraded by hydrolysis, the degradation of polylactide is divided into two stages: the nonenzymatic melting of ester groups and the random scission of low-molecular-weight polymers by microorganisms to produce carbon dioxide and water [58,59].

### 2.4. Improvement of PLA Properties

Although polylactic acid has good biocompatibility and stretchability, the properties of this seemingly promising polymer are not perfect and still need to be improved [60]. For example, PLA is extremely hydrophobic. This property makes it unsuitable for drug delivery, and its low impact toughness causes it to have certain disadvantages as a material for implants in the field of bone transplantation in a high-mechanical-strength environment [61]. However, certain methods can be employed to effectively improve the above deficiencies of polylactic acid and make it more suitable for application in various fields. As a nanocomposite, the properties of PLA can be improved by adding modified additives during synthesis, or by directly blending it with other polymers. Copolymers with low Tg and flexibility may be more suitable for use in implantable medical devices and drug-delivery systems [42,62,63]. Therefore, increasing attention is being devoted to improving the low-temperature performance and malleability of polylactic acid. For example, polyglycolic acid has a high melting point (228 °C) and high Tg (37 °C) [64]. Its addition can lead to an amorphous polymer with a lower Tg, and this polymer is compatible with pure poly. Compared with l-lactide, polyglycolic acid has a higher hydrolysis rate due to its increased hydrophilicity. Therefore, the copolymer of ethylene glycol (5) and lactide, which has increased hydrophilicity and flexibility, has long been used commercially in biocompatible surgical sutures. Furthermore, since the ester bond of polylactic acid is sensitive to enzymes, it can easily be catalyzed and hydrolyzed, leading to the disadvantage of too-fast drug release in drug-delivery systems. The copolymer consisting of LA and glycolic acid in a ratio of 2:23 known as VIRYL (Ethicon Inc.) [29] improves the controlled release of the drug. The copolymer formed with ε-caprolactone (6) changes the properties from rigidity to elasticity. The Tg of poly(ε-caprolactone) is −60 °C, and its melting point is approximately 59.5 °C. When the monomer is combined with pure l-lactide, l-PLA blocks are formed that exhibit high flexibility and crystallization with a high melting point. However, to synthesize tough polymers with favorable low-temperature properties, these blocks must be sufficiently large. Grijpma used a 1:1 ratio to synthesize a block with a longer sequence, a Tg of −39 °C, and a tensile strength of 18.2 MPa. Compared with the ε-caprolactone monomer, the monomer possessed increased tensile strength ranging from 0.6 to 48 MPa; an improvement exceeding 400%. Using tin octoate as a catalyst, copolymerization of lactide with different amounts of BMD (15), which is a polymer with carboxyl functional side chains, can be used to prepare polylactic acid.

A simpler modification method is to blend two different polymers. The blending effect is amazing. The blend shows superior physical and mechanical properties than those of the original polymer [65]. Another method is to add plasticizers. Some plasticizers, such as low-molecular-weight citric acid, succinic acid, tartaric acid, and oxalate, are blended with PLA to change its mechanical and thermodynamic properties. There are many reports on using citrate and montmorillonite (MMT) to change toughness and plasticity [66,67,68,69]. The addition of plasticizer reduces the Tg of PLA by 26 °C. Adding PEG (polyethylene glycol) to the polylactic acid/MMT blend produces more agglomerated structures, and the elongation at break of the material remains below 5% [69]. In order to change the biocompatibility of PLA via the blending method, starch can be used [29]. This low-cost method is simpler than synthesizing copolymers, and it also changes the mechanical properties. The accretion of starch leads to a reduction in tensile strength and elongation, and enhanced water absorption, which has advantages and disadvantages, such as an increase in the brittleness of the material. The addition of plasticizers can make up for this shortcoming. The use of polyether can develop a good hydrophilic, nontoxic, biodegradable, biocompatible, and flexible polymer. Zhu and coworkers used a 1.0:1.2 molar ratio of lactide and ethylene oxide to synthesize polymerides due to their different hydrophilicity levels, making it possible to control degradation and drug release. The above-mentioned block copolymers usually have the thermal properties of the two substances of which they are constituted. Copolymers formed by polyethylene oxide (PEO) and PLA have the same mechanical properties as those of the aforementioned ε-caprolactone and PLA copolymers. However, due to the addition of PEO, the hydrophilicity of this polymer is relatively high, which leads to accelerated hydrolyzation and molecular-weight degradation. Kimura also discovered that using polypropylene oxide instead of PEO makes it possible to obtain ABA block copolymers with higher molecular weights.

The above methods change the physical and rheological performance of polylactic acid. The crosslinking method influences the thermal and rheological properties of the material without affecting its mechanical properties. The implementation of crosslinking is very easy; the multifunctional monomer can be added during the polymerization process. For example, when crosslinking with 5,5′-bis(oxepane-2-one) (bis(E-caprolactone), so that the PLA solvent swells less, the gel fraction is greater than 96%. Furthermore, a small amount of crosslinking agents also causes coagulation, and glue fractions greater than 80% can be used to produce impact-resistant machined products. Polymer free-radical recombination is another effective method for inducing crosslinking in polymers. The formation of free radicals by peroxides has proven to be an efficient and controllable way to produce different levels of crosslinked PLA [9].

As mentioned above, the polymer can be degraded by ordinary hydrolysis of the ester bond, without enzymes. The degraded hydrolysate is nontoxic and is metabolized in the human body, subsequently being eliminated from the body through urine. Polylactic acid has good biocompatibility and degradability, which has led to its being widely used in the medical field [40,70,71,72]. In addition to being used in sutures, PLA is a bioabsorbable material and is widely used in orthopedic surgery [32,73]. Fixed devices such as dissolvable suture nets and absorbable steel plates are good examples of its medical applications. Compared with traditional metal-steel plates, PLA does not corrode bones after being implanted in the human body. Because it can be absorbed into and degraded within the body, time and money are saved, while simultaneously reducing the need for secondary operations. PLA also has great potential as a drug-delivery carrier. It can effectively control the release of drugs at the lesion site and decrease the side effects caused by drug bursts; it can also be modified to accurately deliver drugs to lesion sites, reducing side effects and improving bioavailability. This undoubtedly provides a good platform for drug-delivery systems. In the next section, we focus on applying PLA in the biomedical field [74].

## 3. Application

Good biocompatibility is an important reason for the wide use of PLA in the field of biomedicine. The intermediate lactic acid product can be metabolized by the human body and is nontoxic and harmless. Its degradation can be used for drug release. By controlling the metabolism rate of the carrier in the body, it is possible to ensure the effective concentration of the drug while reducing side effects, which gives it obvious advantages for use both in drug delivery and as a material for implants. Good biocompatibility also ensures that there is no inflammatory reaction in the surrounding tissue due to rejection after it enters the human body [75,76,77].

### 3.1. Tissue Engineering

Blood transfusions carry with them the danger of viral infection; for this reason, research into artificial blood has attracted a great deal of attention. The main component of blood is red blood cells, which transport oxygen [78,79,80,81]. Microcapsules can encapsulate animal hemoglobin [82,83]. Oxygen enters the microcapsule through the membrane wall and is combined with hemoglobin so that that the hemoglobin is able to transport oxygen. This method of embedding can also prevent animal hemoglobin from being produced in the blood. The antigen–antibody reaction extends the half-life. Some studies used liposomes to encapsulate hemoglobin. However, liposomes have a low entrapment rate and are unstable when entering the blood circulation. They easily release hemoglobin and have a short half-life [84,85,86]. Su et al. found that, when polylactic acid was used as the membrane material, the embedding rate of hemoglobin was very low, only 7.9%. When using a polylactic acid–polyethylene glycol copolymer, the embedding rate reached 90%. Additionally, when preparing the microcapsules, since the inner and outer water phases are both aqueous solutions, the hydrophilic polyethylene glycol block stretched toward both the inner and outer water phases. The PEG stretched toward the inner water phase was biocompatible. It had good properties and a certain protective effect on hemoglobin, and helped maintain protein activity during storage [87,88,89].

### 3.2. Drug Delivery

PLA can be degraded into LA in and metabolized by the human body. It is a material approved by the FDA for biomedical use. In the 1970s, Yolles et al. used PLA for drug-delivery research, and prepared a PLA complex containing naltrexone. The in vitro release rate was 67% at 35 days. In vivo experiments proved that the complex had a blocking effect on morphine. Ruan et al. used PLA copolymers to prepare PLA–PEG–PLA microspheres containing paclitaxel [90,91]. The prepared microspheres were uniform in size and had a porous structure inside that promoted drug release. In vitro release reached 49% in one month, prolonging drug action time in the body and improving its efficacy. This sustained-release drug-delivery system avoids inconsistent local concentration due to drug release, thereby enhancing the therapeutic effect. Compared with traditional dosage forms, this slow-release “smart” drug-delivery system has many advantages: reducing the side effects of drugs on the gastrointestinal tract and avoiding high local drug concentrations within a given period, which may lead to allergies or even poisoning. Additionally, degradable materials can be degraded in specific environments, meaning that external polymer materials could be modified to achieve the controlled release of drugs [92,93,94].

**Figure 4:**
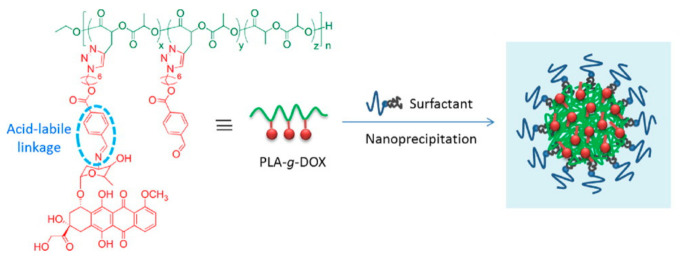
High drug loading and pH-responsivity PLA-*g*-doxorubicin (DOX) nanoparticles [99]. Reprinted from Cheng with permission from Express Polymer Letters.

For some specific diseases such as tumors and tuberculosis, therapeutic drugs can possess high toxicity and extensive side effects. In addition to the killing effect at the lesion site, they also cause great damage to normal tissue. At the same time, the process of treatment requires multiple and frequent administrations [95]. Therefore, targeted drug systems are worthy of study [96]. This kind of coating material, combined with target selection achieved through the affinity of the functional group, can quickly target the lesion site with the drug, improve its bioavailability, and reduce its toxic side effects and toxicity to other tissue types [97,98]. However, the polymer must be chemically modified to be more useful for targeting or eliciting a stimulatory response. As shown in Figure 4, a kind of pH sensitive linkage was designed to modify doxorubicin loaded PLA nanoparticles which make it easy to disintegrate in tumor microenvironment. Therefore, modified polylactic acids such as PLGA (Polylactoglycolic acid) are usually used in drug-delivery systems. Ferric oxide plays an important role in drug-delivery systems for magnetic nanoparticles. Fang et al. used PLGA to embed ferroferric oxide, and then encapsulated doxorubicin (DOX) inside to form a nanocomposite carrier. This carrier maintains high sensitivity to the external magnetic field, thus achieving magnetic targeting by the carrier. This medicine also has good antitumor activity [99].

Thermosensitive gels also play an important role in delivery. Most thermosensitive gels have hydrophilic ends composed of PEG and hydrophobic ends composed of degradable polyesters, such as PLA and PLGA, polyglycolic acid (PGA), and polycaprolactone (PCL). Temperature-sensitive gels based on a polylactic acid composition can be divided into block components of diblock, triblock, multiblock, star, and other gel systems. The phase-change phenomenon during the heating process takes place as described in Table 1 [100,101].

**Figure 5:**
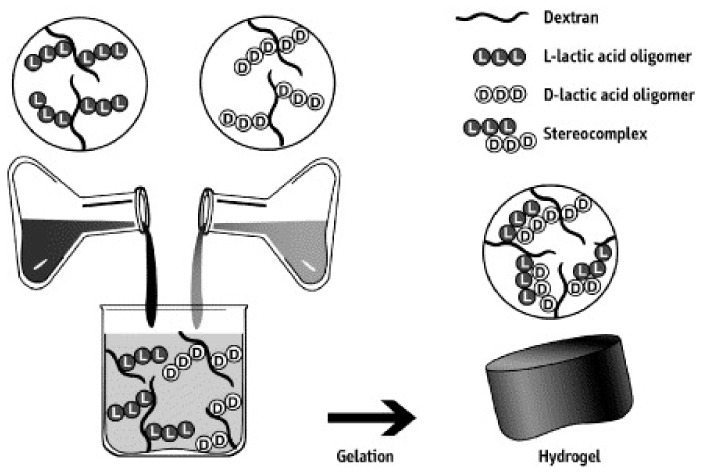
Schematic illustration of the concept of stereocomplex hydrogel formed by mixing aqueous solutions of bio-polymers containing enantiomeric oligo(lactic acid) [125]. Reprinted from Nostrum with permission from Express Polymer Letters.

The earliest report on PEG–PLLA diblock temperature-sensitive gels in nature led temperature-sensitive physical gels to become a research hotspot. Triblock copolymer PEG–PLLA–PEG takes PLLA as its center and exhibits a gel–sol phase transition during the heating process. Multiblock copolymers can improve the mechanical properties of gels. Compared with amorphous (PEG–PDLLA)_n_ multipolymers, stereoregular (PEG–PDLLA)_n_ multiblock copolymers have a lower phase-transition temperature, and higher gel storage modulus and mechanical strength. Star-shaped micelles formed by PEG-*g*-PLGA have smaller hydrodynamic radius and lower viscosity than those of linear copolymers with the same molecular weight. The star-shaped structure is conducive to gelation [122]. The eight-arm PEG-(PLLA)_n_ star copolymer prepared by Nagahama had a sol state at room temperature, and formed a gel as the temperature was increased. Degradation time in the phosphate buffer system exceeded one month. All these factors make it an ideal sustained-release delivery model [123,124]. There is an example of a gel drug delivery system as shown in Figure 5.

### 3.3. Implants

Bone damage caused by natural disasters, traffic accidents, sports trauma, bone-tumor resection, congenital bone diseases, metabolic osteoporosis (OP), and a variety of bone repairs and fractures is a major issue that threatens human health [126,127,128,129]. The repair of bone damage caused by OP-induced fractures or other problems is a major problem in the 21st century [130,131,132,133]. In order to address this, scaffold materials based on the biomimetic human extracellular matrix (ECM) are considered to be the most promising biomedical materials for use in bone-tissue regeneration and repair. With respect to biological safety and the characteristics of metabolites, polylactic acid is considered to be an advantageous material in comparison to copolymers such as polyglycolic acid and polycaprolactone [134,135]. Although the properties of PLA are not perfect, by blending it with hydroxy phosphate lime (HA), the scaffold can be given osteoinduction and bone conduction properties. Additionally, the elastic modulus of the scaffold can be improved. Quirk et al. used the adsorption method to incorporate RGD-immobilized polylysine into PLA. All of these physical modification methods are unstable when compared to the chemical modification of biologically active factors [136]. Therefore, with a view towards the long-term application of tissue-engineering materials, chemical modification methods have attracted a great deal of attention. Some researchers used polyethylene glycol, polyglycolic acid, and PLA to copolymerize and improve the biological functionality of polylactic acid. By copolymerizing lactide and lecithin, Iwasaki successfully introduced the phospholipid choline molecule (MPC), a cell-active biological membrane component, into the PLA structure. Results showed that the phospholipid choline molecule reduced protein adsorption and platelet adhesion on the material surface. The addition of phospholipid choline molecules inhibited the adhesion of white blood cells. Yasuhiko’s award-winning phospholipid choline molecule was copolymerized with 2-ethylhexyl acrylic acid (EHMA) and then blended with PLGA, reducing the inflammatory response of the material. Junji et al. copolymerized MPC-containing double bonds with *n*-butyl methyl methacrylate and PLA. The amount of protein adhesion on the surface of the material was reduced, which enhanced the adhesion of cells and materials. Adjusting MPC and PLA makes it possible to control the adhesive growth ability of cells. Therefore, the biomimetic modified polylactic acid materials described above effectively improve biocompatibility and impart new biological activities to the material, which can be useful for broadening the applicability of polylactic acid biomedical materials for use in implanting medical devices in the body, especially with respect to built-in devices with good blood compatibility. This is of significant scientific importance, and possesses great value. Drug-eluting stents are currently available on the market for clinical applications. Drugs are adsorbed onto the stent. Several medical device companies developed PLA-based stents and put them into production [137,138]. Nowadays, 3D-printed biodegradable scaffolds have good biocompatibility, and promote angiogenesis and osteogenesis, thereby benefiting bone development and reconstruction. Some studies evaluated PLA scaffolds made using 3D-printing technology, finding that it results in less inflammation, making it a promising material for repairing bone damage [139,140].

## 4. Conclusions

Completely degradable PLA material solves the white-pollution problem caused by petroleum-based materials, and has become the top priority in the sustainable development of science and technology. Because PLA possesses problems such as brittleness, poor mechanical properties, slow crystallization speed, and low crystallinity, it is severely restricted in many aspects, especially with respect to blown films. In order to overcome the shortcomings of PLA, scientists modified it using physical or chemical methods and prepared a series of ideal materials with good biodegradability, flexibility, and heat resistance. Along with its low price, promising research results in this area have guaranteed and expanded the application of PLA in many fields, providing a platform for production and manufacturing in the fields of industry, 3D printing, advanced technology, and the biomedical field.

Stents based on polylactic acid have been commercialized. However, its application in drug delivery still faces great challenges in the field of biomedicine. Although there have been many studies using polylactic acid to synthesize nanocarriers for the treatment of tumors and other diseases, none of the nanoparticle has been successfully marketed in actual production. Side effects and toxicity are essential in this field among the many reasons for this phenomenon. The size, position and shape of the nanoparticles are all important features, and positively charged nanoparticles can cause certain cytotoxicity, oxidative stress, and even DNA damage. The excessive of reactive oxygen species (ROS) can cause local inflammation. In addition, hemolysis tests are required before nanoparticles are tested in vivo, as slight red blood cell damage may be caused by PLA nanoparticles. The microenvironment of the disease site is complex. For example, the pH levels in tumors and inflammation sites are often lower than normal. Some researchers use this feature to modify PLA. Nanoparticles prepared with PEG–PLA are stable in a neutral environment, but their degradation is accelerated in an acid environment. Thus, the role of targeted drug delivery is realized. However, such seemingly ideal nanoparticles are difficult to achieve in vivo for the complex environment of blood makes nanoparticles facing huge challenges. Brittle nanoparticles will be destroyed by enzymes after entering the blood circulation, or electromagnetically adsorbed and aggregated with proteins, which will trigger immune rejection. Therefore, commercialization of nanoparticles and their application to the human body still face great challenges.

In the process of commercialization of polylactic acid, the price is a key issue to be solved. To solve the problem of the price of PLA, it is necessary to start with the production of raw materials by improving the production efficiency of lactic acid and obtaining high-quality lactic acid at low cost. A further improvement in the production process of lactic acid is required due to the low-cost materials and high-performance microorganisms. It can be seen that the optimization of the production process of lactic acid is also a challenge for the bulk production of polylactic acid.

## Figures and Tables

**Table 1:** Various types of thermo-induced physical hydrogel.

Type of Hydrogel	Copolymer	Phase Transition	Ref
diblock	PEG–PLA	gel–sol	[102]
	PEG–PLLA	gel–sol	[102,103]
	PEG–PLGA	gel–sol	[103,104]
	PEG–PLLA/PEG–PDLA	gel–sol	[105,106]
triblock	PEG–PLGA–PEG	sol–gel–sol	[107,108]
	PEG–PLLA–PEG	sol–gel–sol	[102,103]
	PLGA–PEG–PLGA	sol–gel	[109,110]
	PLA–PEG–PLA	sol–gel	[111]
	PLLA–PEG–PLLA	sol–gel	[112,113]
	PDLA–PEG–PDLA	sol–gel	[114,115]
graft	PEG–g–PLGA	gel–sol	[116]
multiblock	(PEG–PLA)_n_	gel–sol	[117]
	(PEG–PLLA)_n_	gel–sol, sol–gel	[118,119]
star shape	PEG–(PLLA)_n_	gel–sol	[120]
	PEG–(PLLA)_8_/PEG–(PDLA)_8_	gel–precipitate	[121]
